# Thoracic Intervertebral Disc Herniation Associated With Chronic Anabolic Androgenic Steroid Use: A Case Report

**DOI:** 10.7759/cureus.62438

**Published:** 2024-06-15

**Authors:** Nathaniel A Cleri, Jason Zhang, Gurinder Singh, Charles B Mikell, Courtney Pendleton

**Affiliations:** 1 Neurosurgery, Stony Brook University, Stony Brook, USA; 2 Internal Medicine, Mount Sinai West, New York, USA

**Keywords:** thoracic disc herniation, intervertebral thoracic disc herniation, spine, collagen degradation, anabolic androgenic steroids, thoracic intervertebral disc herniation

## Abstract

Anabolic androgenic steroids (AAS) are relatively cheap and accessible medications, commonly used by athletes and bodybuilders for performance enhancement and muscle growth stimulation. AAS usage has been associated with musculoskeletal injuries, such as tendon and ligament ruptures, and numerous other detrimental health effects. Despite these risks, individuals continue to self-administer these drugs in supraphysiologic doses. Here, we present a case of a male bodybuilder with chronic AAS use who developed a spinal thoracic intervertebral disc herniation requiring decompression and fusion. We use this case to highlight a severe potential risk associated with chronic AAS abuse and review the current literature on the biochemical, physical, and physiologic mechanisms linking chronic AAS use, weight-bearing exercise, and the risk of musculoskeletal injuries such as intervertebral disc herniations.

## Introduction

Thoracic disc herniations represent only 0.25-0.75% of all herniated discs [1]. When they occur, 75% are in the more mobile thoracic spine below T8 [2,3]. The peak incidence is between the third and fifth decades of life (80%), and only 25% are traumatic in origin [1,3]. Pain, sensory disturbances, and motor changes are the most common presenting symptoms, with refractory pain and progressive myelopathy serving as primary indications for neurosurgical intervention [2]. Despite these indications, herniated thoracic discs requiring surgery are rare [3]. We present, to the best of our knowledge, the first case of a 45-year-old male bodybuilder with chronic anabolic androgenic steroid (AAS) use who developed a thoracic disc herniation requiring surgery. Overall, this case report aims to review how AAS leads to collagen degradation, serving as a likely causative factor for intervertebral disc herniation.

## Case presentation

A 45-year-old right-handed male bodybuilder with a past medical history of pulmonary sarcoidosis (last flare 15 years ago), polycythemia secondary to recreational AAS use, occasionally requiring therapeutic phlebotomy, and recent COVID-19 infection (eight days prior), presented to the ED for one week of worsening lower extremity weakness and falls. His lower extremity weakness was described as numbness and tingling to his toes bilaterally. He described feeling clumsy at home, further characterized by buckling at the knees, difficulty lifting his feet, and directing his feet to walk, leading to multiple falls. When asked about potential trauma, he recalled acutely straining his back while performing overhead manual labor. The next day, while again performing extensive overhead work, he further aggravated this back pain. Six weeks later, he visited the gym to exercise where he felt an abnormal sensation in his foot after walking on a treadmill and intensely using an abdominal crunch machine. The patient experienced further weakness in his legs during his workout the next day; when he attempted to walk on a treadmill, he was unable to do so without holding himself up. This and his additional falls at home led him to seek medical care. He had no surgical history. His home medications included ibuprofen 800 mg daily, diclofenac topical gel, and a vitamin D supplement. He denied tobacco, vaping, alcohol, or other illicit drug use. He had no allergies. His family history was noncontributory. He presented to the ED at the advice of two outpatient neurologists he consulted for the same chief complaint.

At presentation, he was hypertensive (systolic blood pressure = 185 mmHg; diastolic blood pressure = 125 mmHg). All other vital signs were within normal limits. His physical exam was notable for slightly decreased motor strength when flexion and extension were assessed at the hip and knee bilaterally. Motor strength testing may have been confounded by the patient’s exceptional baseline strength. Pain sensation was decreased in his feet bilaterally. He had no sensory level on his trunk. He displayed no abnormal movements or pronator drift. His upper extremities were areflexic bilaterally. He displayed no Babinski or Hoffman reflexes and no ankle clonus. Vibratory and joint position sensations were intact throughout. His coordination was intact. His gait was wide-based, slightly spastic, and unsteady. The remaining physical exam was unremarkable. Routine labs were notable for increased glucose (109 mg/dL) and hematocrit (64.2%), and he was SARS-CoV-2 positive, tested via polymerase chain reaction. The remaining indicated lab tests were within normal limits.

Weight lifting and anabolic androgenic steroid use interview

The patient was also interviewed in-depth regarding his exercise and performance-enhancing drug use regime. He began lifting weights at the age of 15. He exercises seven days per week. His total weekly volume consists of one hour each of back, bicep, chest, triceps, deltoid/trapezius, and leg muscle resistance-based exercises. He mainly uses free weights and machines in his exercise routine. He incorporates a Smith machine, hack squats, and safety bar squats in his leg routine. He has only performed the deadlift four times in his life. He also reports using an abdominal crunch machine on a highly regular basis.

At 18 years old, he began taking testosterone isocaproate (Sustanon) 250 mg daily and metandienone (Dianabol) 10 mg daily for 17 years straight. In 2006, the patient’s blood testosterone levels measured 1500 ng/dL on one occasion (normal range: 300 to 1,000 ng/dL). Between 2018 and 2020, the patient reported self-administering trenbolone (dose unclear) every other day. In 2020, the patient self-administered one intramuscular injection of testosterone isocaproate weekly (dosage not recalled). On his most recent testing, his testosterone level was 250 ng/dL (normal range for males aged 40-44: 350-473 ng/dL) after taking trenbolone [4]. He states that he has decreased his anabolic steroid use over time. Table 1 displays notable vital signs and lab values for our patient.

**Table 1:** Patient's notable vitals and lab values.

	Patient’s lab value	Normal value
Systolic blood pressure (mmHg)	185	<120
Diastolic blood pressure (mmHg)	125	<80
Glucose (mg/dL)	109	70-100
Hematocrit (%)	64.2	40-54
Testosterone (ng/dL)		
2006	1500	300-1000
Recent testing	250	350-473

Imaging

**Figure 1:**
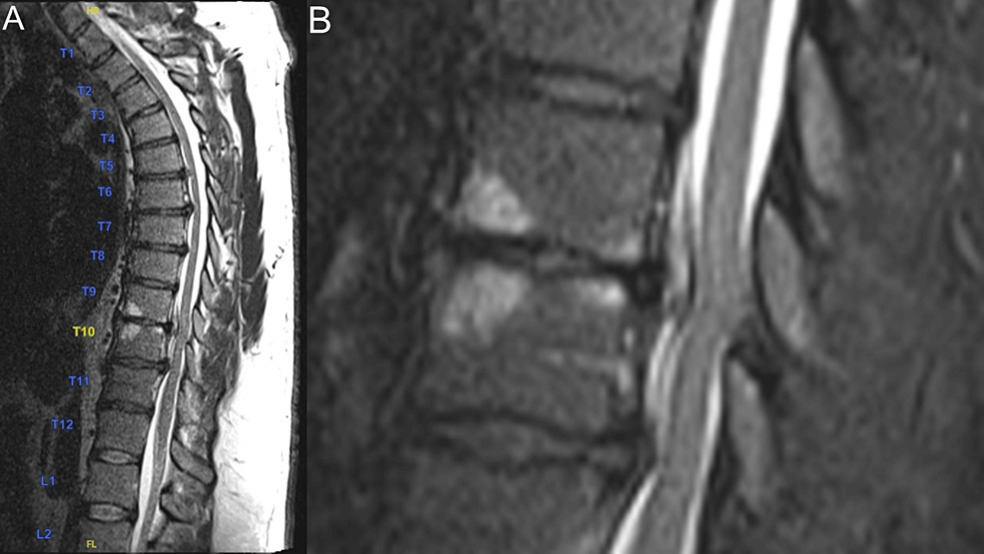
T2 sagittal thoracic spine MRI. (A) T2-weighted midsagittal spine MRI. (B) T2 sagittal inset T9/10/11 1.5x zoom.

**Figure 2:**
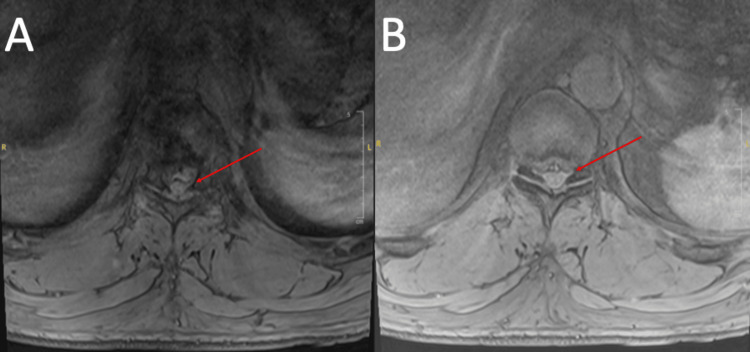
T2 axial thoracic spine MRI. (A) T9/T10. (B) T10/T11. Red arrows indicate the area of interest.

Thoracic spine MRI (Figures 1, 2) was most notable for direct spinal cord compression at T9/T10 from a craniocaudally directed intervertebral disc herniation and a superimposed disc bulge. This caused adjacent spinal cord edema, acute myelopathy, and compression of the T9 nerve roots bilaterally. There was also ventral spinal cord impingement at T10/T11, severe right foraminal stenosis, and right T10 nerve root compression from disc herniation at this level.

Surgery

**Figure 3:**
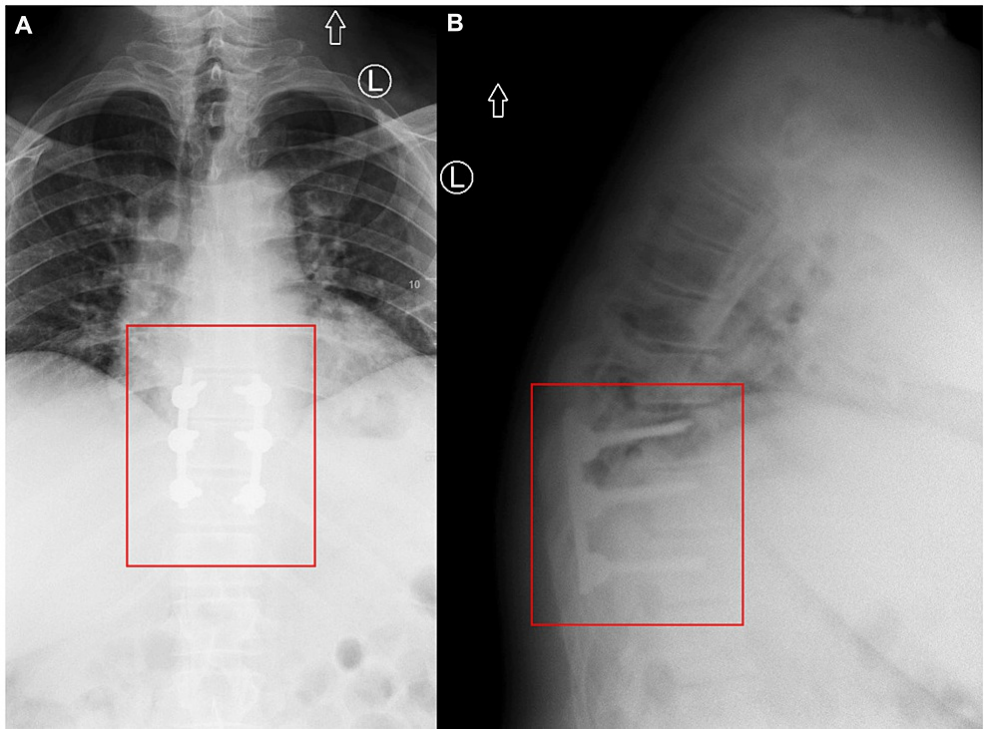
Postoperative thoracic X-rays. (A) Anterior-posterior X-ray showing pedicle screw placement from T9 to T11. (B) Lateral X-ray showing pedicle screw placement from T9 to T11. Red boxes indicate the area of interest.

He urgently underwent an uncomplicated posterior thoracic (T9-T11) laminectomy and instrumented fusion. Total laminectomies were performed at T9 and T10, and a subtotal laminectomy was performed to the top portion of T11. All three levels were instrumented bilaterally. Somatosensory and motor-evoked potentials were at baseline following the operation. He was discharged four days later after a straightforward hospital stay. Postoperative X-rays are shown in Figure 3 demonstrating the T9 to T11 pedicle screw instrumentation.

Two months post discharge, his lower extremity symptoms were nearly resolved and his incision was well-healed with minimal superficial pain. He denied numbness, weakness, bowel and bladder issues, and or perineal sensation changes. He had fully intact muscle strength and sensation throughout his lower extremities with normal reflexes and no clonus. He is now walking normally and denies any falls or traumas. He was subsequently cleared to return to work and full activities.

## Discussion

Despite speculation that excessive AAS use may be related to cervical disc herniation in high-profile bodybuilders, such as former “Mr. Olympia” Ronnie Coleman, few researchers have investigated this topic [5]. To our knowledge, this is the first report of a thoracic disc herniation in a professional bodybuilder using performance-enhancing AAS.

The nucleus pulposus is composed of exclusively type II collagen, while the outer edge of the annulus fibrosis is exclusively type I collagen [6-8]. Moreover, the total collagen composition of intervertebral discs decreases with increased age and use, as accumulated microtears cause erosion and weakening [9-13]. While the prevalence of disc herniation in the general population varies, it has generally been observed that disc herniation incidence is increased in performance athletes (i.e., professional football players, Olympic athletes, and bodybuilders) and individuals using AAS [11,14,15]. Commonly abused AAS include testosterone (Sustanon and related formulations), oxandrolone (Anavar), metandienone (Dianabol), and nandrolone (Deca Durabolin); however, many others are used [16-18]. In addition to testosterone, AAS include synthetic analogs of testosterone that promote muscle growth and increased strength when used in supraphysiologic doses combined with regular resistance training [18,19].

We speculate that the combination of AAS and the abdominal crunch machine led to herniation in this patient. When considering particular exercises that may cause a greater risk of injury in athletes, previous literature describes how different movements can initiate disc herniation or weaken the annulus fibrosis through the accumulation of microinjuries [20]. Specifically, spinal flexion - required for the abdominal crunch machine our patient utilized before his onset of acute pain - is reported to induce translational movement of the nucleus pulposus that may lead to herniation as it pushes against the surrounding wall of the annulus fibrosus. One peer-reviewed study modeling the mechanics of the abdominal crunch exercise supported this argument and reported that excessive intervertebral joint loading during the crunch exercise could increase the risk of back injury [21]. Another study, reviewing the effects of longitudinal use of the abdominal crunch exercise, argues that spinal flexion exercises can be safely performed within a window of “eustress” in which disc microinjury from the exercise is minimal and can be recovered from presumably without disc degeneration and increased risk of future injury [22,23]. However, in patients with abnormalities in collagen metabolism, as in this patient, this exercise may lead to “distress” and chronic disc weakening that could increase the risk of herniation [22,23].

Furthermore, a long history of associations between AAS use and musculoskeletal injuries raises concerns that AAS may directly impair collagen function by interfering with normal remodeling [15,24]. AAS use is associated with increased tendon stiffness and vulnerability to rupture in both human and animal models [24-29]. Some studies suggest that AAS use may disrupt the organization of collagen within tendons causing them to weaken and become injury-prone, while other studies report being unable to find evidence of AAS-associated changes in collagen microstructure [25,28]. A controlled laboratory study using rats demonstrated that AAS treatment may impair tissue remodeling in the Achilles tendon of animals undergoing physical exercise by down-regulating matrix metalloproteinase (MMP) activity (a marker for tendon remodeling), increasing the potential for tendon injury [27]. Specifically, all rats treated with AAS had decreased MMP activity, while the exercise-only cohort had increased MMP activity [27]. Marqueti and colleagues also demonstrated that the “exercise + AAS” cohort had inflammatory infiltration and fibrosis in their tendons on microscopy, suggesting that modulation of MMP activity may drive an inflammatory mechanism by which AAS use potentiates fibrous connective tissue injury [27]. In this report, we speculate that impairment of these repair mechanisms may have led to the herniation.

While we speculate that the combination of AAS usage and abdominal crunch exercise led to thoracic disc herniation in this patient, we acknowledge that this injury may have occurred from the abdominal exercise alone. However, given the patient’s prolonged use of AAS and their known destructive effects on collagen - the fundamental building block of intervertebral discs - we believe AAS abuse was inherent to his herniation. With AAS use rising due to ease of accessibility, further investigation into the prevalence of disc herniations in the AAS-using population is necessary.

## Conclusions

We report the first case of thoracic disc herniation associated with AAS use. We speculate that the mechanism is related to impaired collagen metabolism in combination with chronic stress from an abdominal crunch machine. Further studies on large cohorts of AAS users will likely confirm our view that AAS use increases disc degeneration, in accordance with predictions about how AAS affect collagen.
